# Sunglint imprints of steady subcloud cells anchoring intermittent trade cumulus

**DOI:** 10.1038/s41612-026-01427-3

**Published:** 2026-05-12

**Authors:** Ilan Koren, Orit Altaratz, Yael Arieli, Bar Moisa

**Affiliations:** Department of Earth and Planetary Sciences, Weizmann Institute of Science, Rehovot, Israel

**Keywords:** Climate sciences, Environmental sciences

## Abstract

How can seemingly random, shallow trade-wind clouds be predicted and their climate impact better evaluated? These clouds cool the planet but are hard to represent because they appear sparse and short-lived. We show that, beneath this apparent disorder, the flow is organized as a dense lattice of steady convective cells that tile the subcloud layer. These cells form the dynamic backbone of the cloud field, with their updraft walls serving as persistent launch points for cloud plumes. Spaceborne ocean-sunglint observations reveal a matching 1-2 km cellular texture beneath sparse cloud cover, consistent with the subcloud convective cells’ morphology. Together, the modeled dynamics and sunglint fingerprint reframe trade cumulus as organized from below, suggesting a physics-based route to reduce climate-model uncertainty.

## Introduction

Low-level marine clouds strongly influence Earth’s radiative balance by reflecting incoming sunlight, yet their response to a warming climate remains one of the largest sources of uncertainty in climate-sensitivity estimates^1,2^. A prominent component is shallow, non-precipitating cumulus in the marine boundary layer (MBL), which forms over large areas of the tropical and subtropical oceans^3^. Because these clouds are sparse and short-lived, and difficult to observe systematically, constraints on their organization and variability remain limited. Therefore, global climate models still struggle to represent how spatial organization modulates albedo and surface fluxes^4,5^.

Cloud organization is a key climate factor because it structures boundary-layer circulations, partitioning updrafts and downdrafts into coherent patterns that modulate cloud fraction, liquid water path, and entrainment, thereby influencing albedo and shortwave cooling^6,7^. This spatial structuring also regulates the onset and persistence of precipitation, shapes aerosol-cloud interactions, and modulates surface fluxes, affecting rain distributions and boundary-layer humidity^8,9^. By acting over large areas, these pattern-dependent radiative and microphysical changes alter top-of-atmosphere radiative fluxes and cloud feedbacks, making organization a major target for improved parameterizations in climate models^1,2,10^.

Non-precipitating trade-wind regions exhibit intermittent, plume-dominated scatter cumulus fields. Observations from EUREC^4^A^10^ and earlier campaigns, together with numerical simulation studies, show that mesoscale variability modulates where coherent plumes and clouds appear and how long they persist, highlighting the organization’s effects on the MBL’s radiation and precipitation budgets^6,7,9,11,12^.

The Trade MBL is often divided into several layers, each with distinct dynamical and thermodynamic properties^13,14^. In particular, the subcloud layer, typically well-mixed and relatively homogeneous in potential temperature and humidity, is frequently separated from the cloud layer above it by a transition layer^15,16^. This transition region marks a zone of changing turbulence intensity, moisture fluxes, and temperature gradients. The cloud layer (the cumulus layer) is commonly described as conditionally unstable in the thermodynamic (parcel) sense^17^. Unsaturated parcels may be stable, but parcels that reach saturation can become positively buoyant up to the inversion^18^. Classic mixed-layer/cumulus-layer frameworks formalize this two-layer view using conserved-variable budgets and cloud-base mass flux closures^19,20^.

It was shown that the subcloud layer organizes into long-lived, surface-rooted coherent dynamical features^21,22^ and that these coherent structures carry a large share of vertical transport^23^. The formation of shallow clouds is shown to be dynamically linked to the subcloud coherent structures updrafts, and the strongest, fastest-growing subcloud updrafts in moister patches are most likely to trigger clouds^24^. Large-eddy simulations and field measurements, including aircraft observations and turbulence measurements from instrumented towers, established the geometry and flux budgets of these structures, and tracking frameworks quantified their lifetimes, their coupling to cloud plumes, and the role of coherent subsiding structures^21,25–27^.

Here, we investigate the nature of the dynamical organization, specifically the role of the subcloud layer’s organization of the velocity fields in non-precipitating trade clouds (TrCu), and its relationship to the sparse cloud’s spatial and temporal properties. We do so following recent reports, describing ordered, dense, and steady thermal convective cell structures forming within TrCu cloud fields that operate continuously throughout the system’s lifetime^28,29^.

## Results

### Vertical profile analysis

To address this challenge from a numerical perspective, we simulated a non-precipitating field of small TrCu clouds forming east of Barbados^18,30^, using a state-of-the-art large-eddy simulation (LES) by the SAM model^31^ coupled with a Spectral Bin Microphysics (SBM) scheme^32^ (see “Methods”). LES models are the primary tool for generating realistic cloud fields, providing high-resolution insights into turbulent processes. The MBL is capped by an inversion above the height of ~1500 m, and the cloud base is located around ~600 m. We focus on the properties of the velocity fields as a direct measure of the field’s dynamics.

**Fig. 1:**
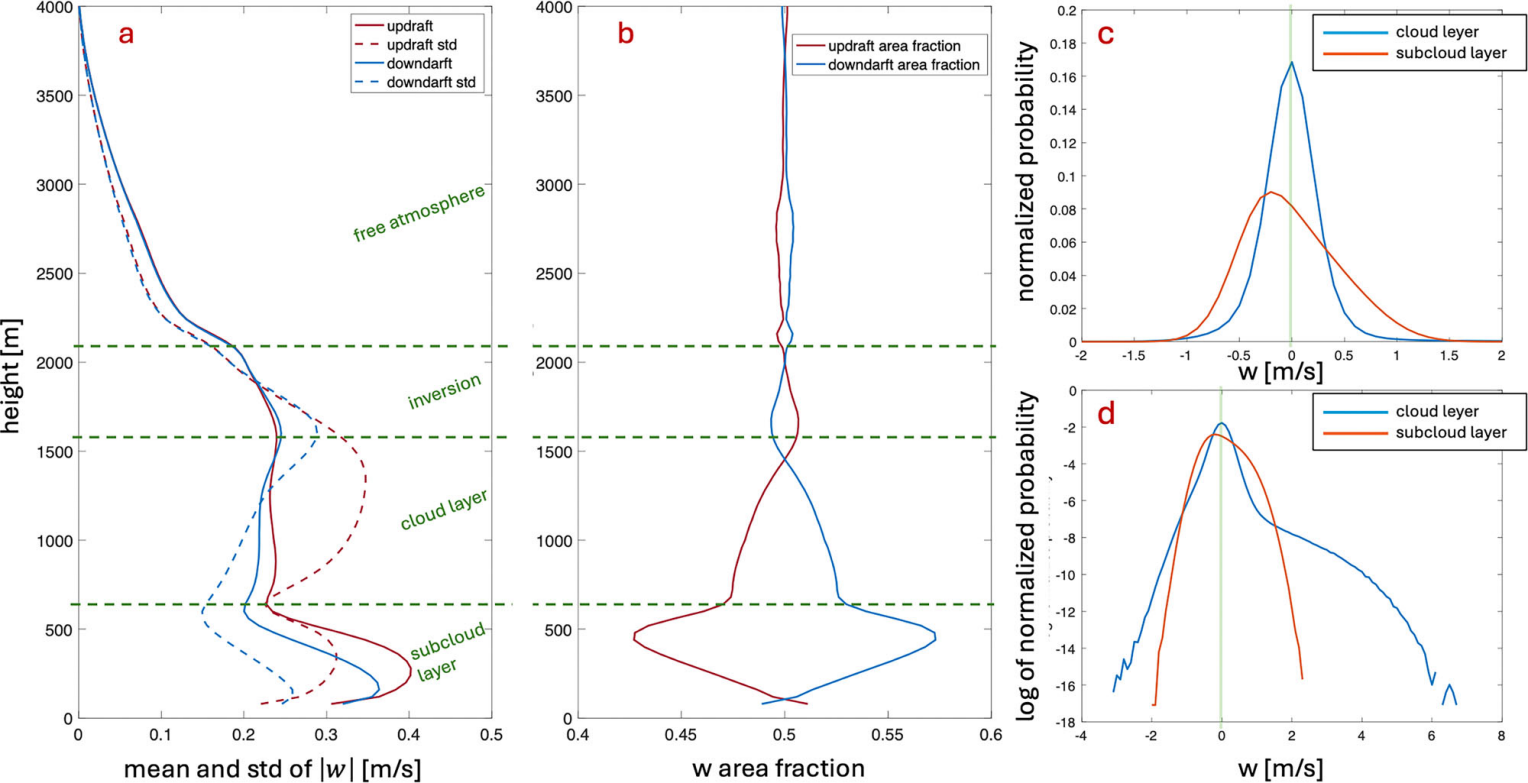
Statistics of vertical velocity across the marine boundary layer. The figure’s vertical structure in **a**, **b** is divided into four horizontal layers: (1) lower MBL subcloud layer, (2) cloud layer, (3) inversion layer, and (4) free atmosphere. Distinct statistical properties are shown for each layer. **a** The mean magnitude of *w* and standard deviation along the updraft *w*_*u*_ and downdraft *w*_*d*_ areas of the field. **b** The horizontal partitioning of the area to downdrafts (blue) and updrafts (red). Note, (1) in the lower part of the profiles, *w*_*u*_ > *w*_*d*_, (2) the almost constant values of the averaged magnitude of the velocities in the cloud layer, and (3) the standard deviation profiles relative to the means. Also, note that the stronger updrafts (lower **a**) have a smaller area fraction (**b**) such that a perfect mass flux balance is kept throughout the profile. **c** Normalized distribution of the vertical velocities in the middle of the cloud layer (height of 1200 m, blue) and in the subcloud layer (height of 240 m, red). **d** The same histograms but on a logarithmic scale. Note the differences between the bulk (in **c**) and the tail (**d**) distributions.

To set the stage and explore the dynamical properties of the field and how they emerge in each layer, Fig. 1 shows the statistical properties of the vertical velocity field (*w*) for each horizontal layer in the model. In panels a and b, the analyses were done separately for the updraft (*w*_*u*_) and downdraft (*w*_*d*_). We compute statistics over an ensemble of all horizontal grid points over a duration of 100 time steps (1 min apart) during a mature, quasi-steady period of the field. At each height level, we pool all vertical velocity samples from the full horizontal domain (512 × 512) across all 100 times and calculate the mean magnitude of the velocities and standard deviation separately for updrafts and downdrafts. Since the downdraft sign is negative, their absolute values are presented to compare the magnitudes. The vertical profiles in Fig. 1a clearly show a separation in the vertical velocity characteristics between the subcloud and cloud layers. It shows that: (1) *w*_*u*_ > *abs*(*w*_*d*_) from the surface up to the inversion layer, above which their values are low, identical, and almost constant. (2) The peaks of the averaged magnitude of the vertical velocities are around the middle part of the subcloud layer. Above the peak, their values decrease until they reach the cloud base. (3) There is an increase in updraft variance in the cloud layer that is driven by the tail of the vertical velocity distribution, as shown in the histograms in panels c and d. As will be shown next, this tail is contributed by the sparse, intermittent, updraft plumes that are associated with cloud formation. Note that the updraft standard deviation reaches its maximum in the upper part of the cloud layer. (4) The velocities’ statistical properties (means of velocity magnitudes and standard deviations) become almost identical for the updrafts and downdrafts above the inversion base. Figure 1b shows profiles of the area fraction *a*_*u*_ and *a*_*d*_ of *w*_*u*_ and *w*_*d*_. It shows upwards to downwards mass flux balance, such that *a*_*u*_ × *w*_*u*_ + *a*_*d*_ × *w*_*d*_ = 0, for each vertical level. We note that the velocity field evolves slowly in time as the thermodynamic state adjusts. However, over the 100-min window, temporal changes are much smaller compared to spatial variability.

### Lagrangian morphological analysis

**Fig. 2:**
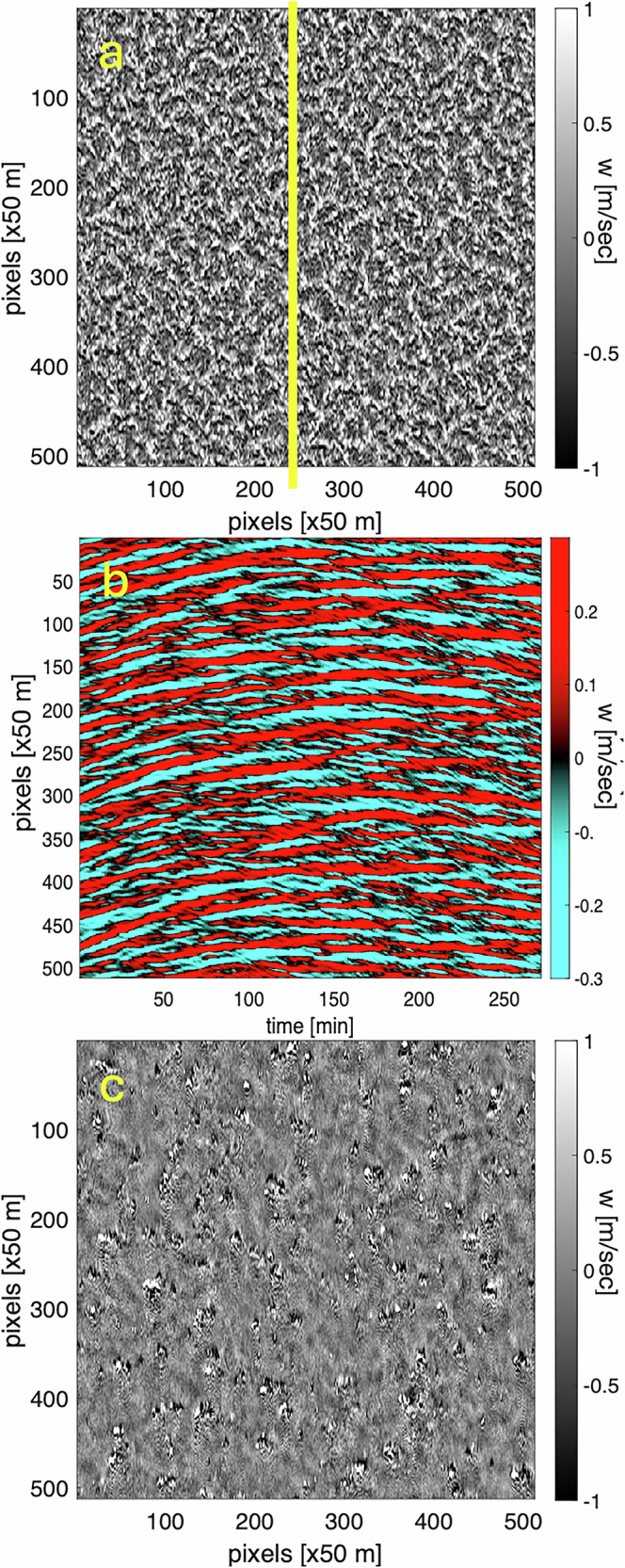
The steady cellular structure of the subcloud layer’s updraft field. **a** A snapshot of the vertical velocity field *w*_240_ in the subcloud layer (at the height of 240 m). **b** The *w*_240_ LHS of the yellow line marked on the updraft field. Red stripes indicate updrafts and cyan downdrafts. **c** A snapshot of the vertical velocity field *w*_1200_ at the middle of the cloud layer.

How does the separation shown in the vertical profiles’ statistics between the subcloud and the cloud layers reflect differences in the morphological properties of the layers’ dynamics? Fig. 2a shows an example of a snapshot of the updraft velocity field (*w*_240_) at the subcloud layer (240 m above sea level). A dense cellular structure that occupies the entire domain is shown, with a characteristic cell diameter as estimated by a 2D spectral analysis (see Supplementary Information Fig. S1)^33^, of ~1.5 km.

Organized cellular convection commonly appears in two idealized morphologies with distinct topology: closed cells, in which updrafts occupy cell interiors, and downdrafts form a connected network along cell boundaries, and open cells, in which downdrafts are concentrated in isolated cell centers while updrafts form a connected network along the cell walls. This open/closed distinction is most often discussed for stratocumulus-topped boundary layers^6,34^, where the horizontal scales are typically larger (order 10 km), and the circulation spans much of the boundary-layer depth. In our subcloud layer, the organization exhibits an open-cell topology: positive vertical velocity (updrafts; light shades) forms a connected lattice outlining cell boundaries, whereas negative vertical velocity (downdrafts) appears as isolated patches near cell centers. In stratocumulus, open-cellular states are frequently linked to precipitation-dynamics feedbacks and associated mesoscale variability^8,35^. By contrast, the non-precipitating trade-wind cumulus regime considered here is not primarily governed by rain-driven oscillations, consistent with the absence of precipitation in our simulations.

To investigate the temporal and spatial persistence of the dynamical structures, we perform a Lagrangian correction^36^ to account for advection in the lower atmosphere^37^ (see “Methods”). After the Lagrangian correction of the subcloud layer, at each time step, we select a stripe at the center of the vertical velocity field (marked in yellow in Fig. 2a) and insert it according to its corresponding time into a Lagrangian Hovmöller Space (LHS). The LHS provides a measure of the selected line’s time-space evolution^28,29^. In this framework, a bright feature, e.g., updrafts along the cell walls, or a dark feature marking the downdraft area in the cell’s center, sampled by the selected line (the yellow vertical line marked in Fig. 2a), is mapped into a horizontal red or cyan line in the LHS (parallel to the time axis), with the line length corresponding to the feature’s lifetime. The LHS of the *w*_240_ field in Fig. 2b shows that dynamical features crossed by the selected stripe produce horizontal lines whose lifetimes are comparable to the entire 5-h duration of the run shown here. Although the LHS lines are not perfectly horizontal due to imperfections in the Lagrangian correction and small-scale shifts of the cells^29^, the *w*_240_ features are clearly identifiable.

A different story emerges when exploring the vertical velocity field in the cloudy layer. Figure 2c shows the updraft velocity field at the middle of the cloud layer (*w*_1200_) (1200 m above sea level). The velocity field appears to be less organized, with sparse, intermittent, isolated spots of high updrafts and downdrafts, and a background field of much weaker velocities occupying most of the domain. The convective TrCu clouds form around the intermittent high updraft spots. We note that the vertical-velocity levels shown here—240 m (subcloud) and 1200 m (cloud layer)—are representative of the respective layer-interior statistics, chosen to avoid transition regions near the surface, cloud base, and the inversion. The strong updraft spots in the cloud layer represent the location of the forming clouds before the weakening of the updrafts in the dissipation stage^38^. Supplementary Information Fig. S2 shows the link between the vertical velocity field in the cloud layer and the integrated liquid water amount in clouds.

The vertical-velocity histograms in Fig. 1c, d provide an additional view of the contrast between the two layers. They show the distribution of *w* in the mid-cloud layer (1200 m, blue) and in the subcloud layer (240 m, red). The subcloud distribution (red) is broader and weakly asymmetric, with a mode on the downdraft side (negative *w*) and a pronounced positive tail, consistent with an open-cell topology in which narrow updraft walls are stronger but occupy a smaller area fraction than the broader downdraft interiors. By contrast, the cloud-layer distribution (blue) is narrower around the center and closer to symmetric, reflecting predominantly background turbulence away from intermittent plume cores.

To emphasize rare strong events, Fig. 1d replots the same distributions on a logarithmic ordinate. While the subcloud tails decay relatively rapidly, the cloud-layer distribution exhibits substantially heavier tails, especially for large positive *w*, highlighting intermittent, localized episodes of strong ascent associated with plume/cloud initiation. These differences are also captured by the distributions’ moments. Both layers have near-zero mean vertical velocity. The subcloud variance is larger (0.21 m^2^ s^−2^) than the cloud-layer variance (0.14 m^2^ s^−2^) as shown by the differences in the distributions’ width in Fig. 1c. In contrast, higher-order moments that weight extreme (tail) events are much larger in the cloud layer: the third and fourth central moments are *M*_3_ = 0.04 m^3^ s^−3^ and *M*_4_ = 0.13 m^4^ s^−4^ in the subcloud layer, compared to *M*_3_ = 0.15 m^3^ s^−3^ and *M*_4_ = 0.70 m^4^ s^−4^ in the cloud layer, indicating stronger intermittency and heavier tails aloft. This translates to skewness differences of 0.42 vs. 2.90 and kurtosis differences of 2.95 vs. 37.91 between the subcloud and cloud layers.

Taken together, the vertical profiles and the morphology of the velocity field reveal two distinct regimes. The subcloud layer is characterized by stronger, organized motions arranged in a dense, steady open-cell topology. The cloud layer, by contrast, is dominated by a weak background field punctuated by localized bursts of strong ascent associated with cloud formation. The Lagrangian Hovmöller analysis (LHS) further indicates that individual subcloud cells persist for times comparable to the cloud-field lifetime (hours)^39^, whereas individual trade cumuli typically last only O(1–10) min^40^. The subcloud circulation behaves as a quasi-steady convective “machinery,” whereas boundary-layer cumuli are inherently transient: they are initiated by short-lived updraft pulses (plumes) that, above cloud base, gain buoyancy from condensation latent heating but are quickly eroded by entrainment and mixing. In non-precipitating conditions, once the updraft weakens, dilution by dry environmental air reduces liquid water content and buoyancy, accelerates evaporation, and leads to rapid cloud dissipation^41^. This framing motivates the next question: what is the nature of the coupling between the two layers?

### Passive tracers analysis

A steady state in time and space of a dense cellular convective system like the one shown in the subcloud layer implies local continuity between the updrafts and downdrafts. The system must be almost closed, with a well-defined ceiling, where a volume element moves up with the updraft until it reaches the top of the layer, transfers heat, and then diverts and sinks in the downdraft. The discontinuity in the field’s statistics and sharp changes in the morphology of the vertical velocity field suggest that the transition layer between the subcloud and cloud layers acts as a ceiling, isolating the subcloud layer enough to maintain a steady state of cellular convection. To quantify the separation between the layers and what fraction of the convective air volume stays in the subcloud layer and recirculates within the convective cells, we have performed several numerical passive Lagrangian tracer experiments, in which we release 100s of thousands of tracers in random locations within the subcloud layer, when the cloud field is well established (after spin-up)^38^. We let each tracer move according to the 3D velocity field and track each one for 2 h.

**Fig. 3:**
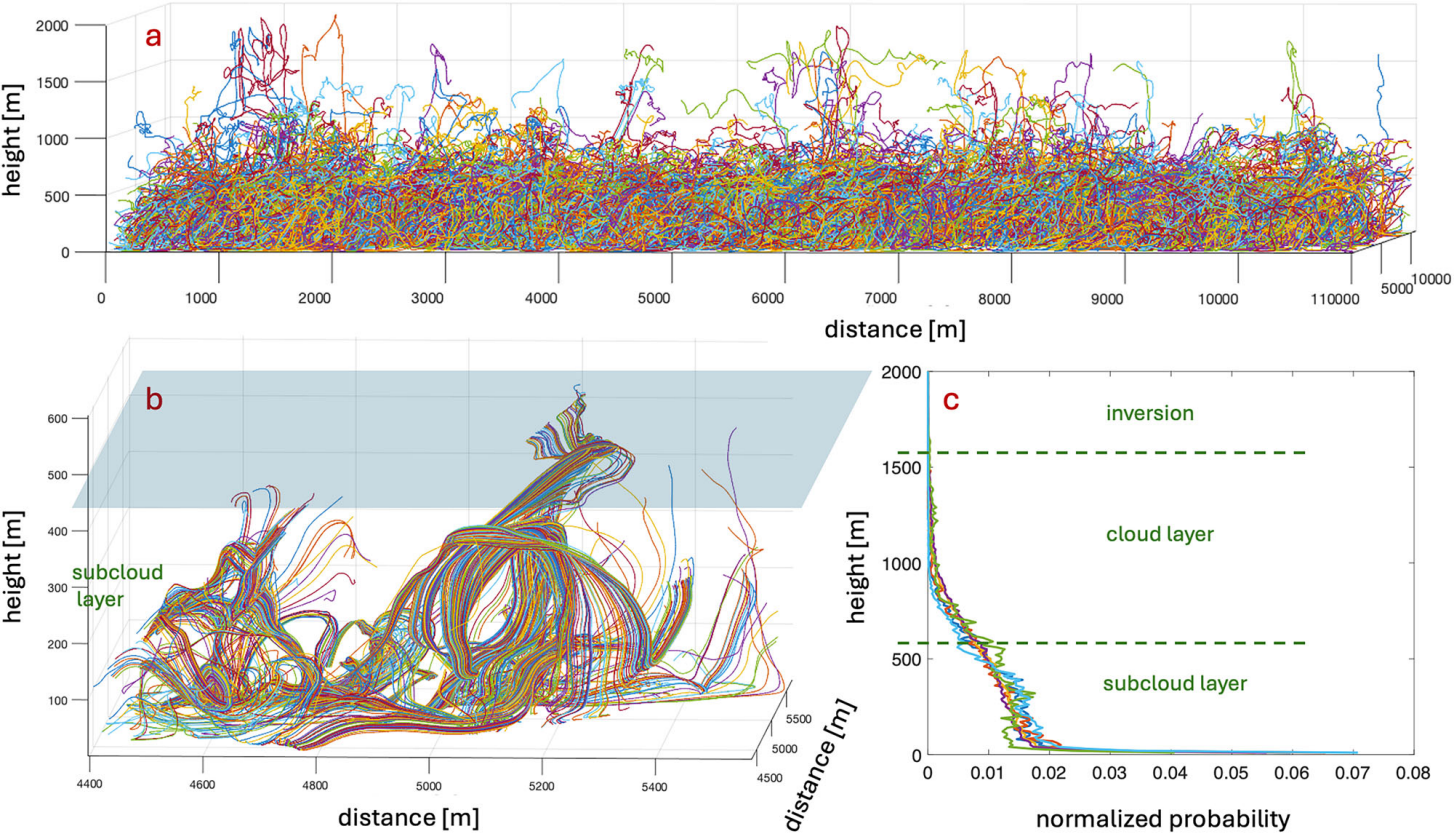
On the link between the cloud and subcloud layers. **a** A side view of one passive-tracer experiment in which the tracers were released randomly within the subcloud layer and were followed for a duration of 2 h. Note how most of the tracers remain within the subcloud layer. **b** Zoom into the center of the field, into the subcloud layer, showing the tracers traveling along one convective cell. **c** Histograms of the passive tracers’ vertical positions after the relaxation time, when no memory of the initial release height remains. Each color represents an experiment in which the tracers were released at different levels within the subcloud layer. Note that apart from noise, the distributions are identical. The distributions indicate that ~15% of the tracers travel into the cloudy layer.

Figure 3a shows a 3D snapshot of a passive tracer field. The tracers were released randomly within the subcloud layer. It shows that most tracers are well confined within the subcloud layer, and the sparse nature of the tracers that do penetrate into the cloud layer. Figure 3b zooms into the subcloud layer in the middle of the field and shows one example of tracers, released at a height of 150 m, traveling along a convective cell. Six passive tracer experiments were conducted, in which tracers were released at varying heights (150–400 m in 50 m increments). Eighty minutes after the initial release of the tracers, their height distribution was invariant, with no memory of the release height. Figure 3c shows the vertical distribution of the passive tracers after an 80 min relaxation time. The different numerical experiments are shown in colors. The distribution shows that ~15% of the tracers penetrated the cloudy layer and their concentration steadily decayed toward the mid-cloud layer.

**Fig. 4:**
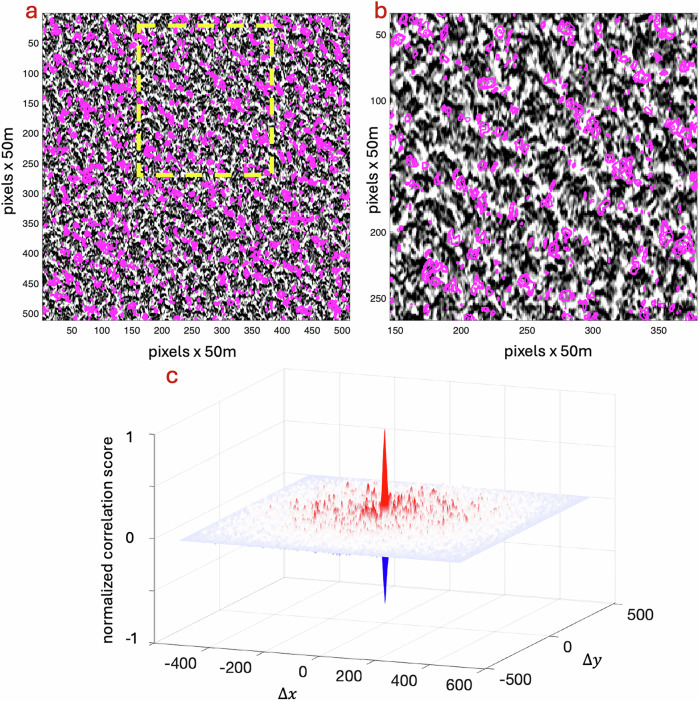
On the link between the convective structure of the subcloud and cloud layers. **a** The grayscale background shows a single Lagrangian-corrected *w*_240_ snapshot taken at the midpoint of that 100-min window (bright = updraft, dark = downdraft; scale as in Fig. 1a). Magenta contours enclose regions where $\overline{{w}_{1200{\rm{m}}}} > 0.3\,{{\rm{m\; s}}}^{-1}$, highlighting cloud-layer’s plumes that align with the subcloud updraft walls. **b** Enlarged view of the yellow rectangle in (**a**), showing how cloud-layer plumes are anchored to the steady cellular pattern of the subcloud layer. **c** Normalized two-dimensional cross-correlation map between one snapshot of the Lagrangian-corrected subcloud field *w*_240_ and the 100 min mean of the Lagrangian-corrected cloud-layer field *w*_1200_. The map is plotted as a function of horizontal displacement (*Δx*, *Δy*) between the two fields. The maximum at zero displacement indicates preferential alignment of strong cloud-layer updrafts with the subcloud updraft-wall lattice, while the sign change at offsets of about half a cell width is consistent with the alternation between updraft walls and downdraft-centered cell interiors.

We observe a steady-state machinery of cellular convective cells operating in the subcloud layer, where most of the air rising from the surface is confined to the subcloud, but a small fraction of the trajectories overshoot and penetrate the cloud layer. The cloudy layer is shown to be composed of relatively weaker vertical velocities and sparse, localized regions with stronger updrafts. What triggers these stronger updraft spots, and how are they linked to the convective steady structure of the subcloud layer? Fig. 4a, b show that most of the updraft spots in the cloud layer, which are likely to represent the locations of the updraft plumes in the field, form above the cells’ updraft walls in the subcloud layer. The contours of the strong localized updrafts in the cloud layer (integrated over 100 min, after Lagrangian correction, to create denser coverage) follow the clear cellular structure of updraft cell walls in the subcloud layer. The contours describe the cells’ edges almost perfectly. It suggests that the limited heat, momentum, and moisture flux that do penetrate into the cloud layer can act as a perturbation, triggering plume formation in the conditionally unstable layer, as indicated by the strong updraft spots and by the clouds (See Supplementary Information Fig. S2). To further assess the persistence of the organized subcloud convection and its spatial correspondence with cloud-layer plume occurrence, we selected one snapshot of the subcloud vertical-velocity field, *w*_240_, and computed its two-dimensional cross-correlation with the 100-min average of the Lagrangian-corrected cloud-layer field, *w*_1200_, starting at the snapshot time. Figure 4c shows the normalized correlation matrix. The map exhibits a clear maximum at zero displacement, indicating that strong cloud-layer updraft regions preferentially occur above the subcloud updraft network and that the subcloud pattern remains sufficiently persistent over the averaging window to be represented by a single snapshot. The correlation decreases rapidly away from the origin, and changes sign at offsets of approximately half a cell width, consistent with the alternation between updraft walls and downdraft-centered cell interiors. Together, these results support a clear spatial coupling between the steady subcloud lattice and the intermittent cloud-layer plumes.

### Subcloud dynamics as shown by delicate changes of the sea surface topography

So far, we have shown, using numerical LES, a dense lattice of steady, convective cells that tile the subcloud layer across the entire domain. Can we find evidence for this steady dynamical organization from measurements? To see it, we have to look at the sunglint zone over the oceans. In satellite remote sensing, sunglint refers to the specular-like bright reflection of sunlight from a water surface that is directly reflected into the satellite sensor. Sunglint usually appears as a streak or a large, bright patch in solar (shortwave) range imagery when the viewing geometry (sun and sensor positions) aligns with this reflection angle. The sunglint area and optical properties depend on the sea surface roughness^42^, and it can be used to estimate surface waves^43^. Over sunglint zones, the strong reflection can saturate detectors and mask other atmospheric, surface, or water-leaving signals (such as chlorophyll, sediment, or aerosols)^44^.

Here, we leverage sunglint’s sensitivity to subtle variations in sea-surface topography, roughness, and facet-slope distribution to detect the imprint of lower-atmospheric dynamics on the ocean surface. In the organized subcloud cellular flow, downdrafts approach the surface and spread radially within cell interiors, producing weak positive pressure anomalies and divergent near-surface flow, whereas the surrounding updraft branches are associated with relatively lower pressure and compensating convergence. These dynamical perturbations modify local wind stress, surface-current divergence and strain, and the growth and dissipation of capillary and short gravity waves, thereby generating spatially coherent variations in sunglint reflectance^42,45,46^. The resulting sunglint texture can therefore be used to diagnose key properties of the subcloud organization, including its cellular structure and characteristic horizontal scales.

**Fig. 5:**
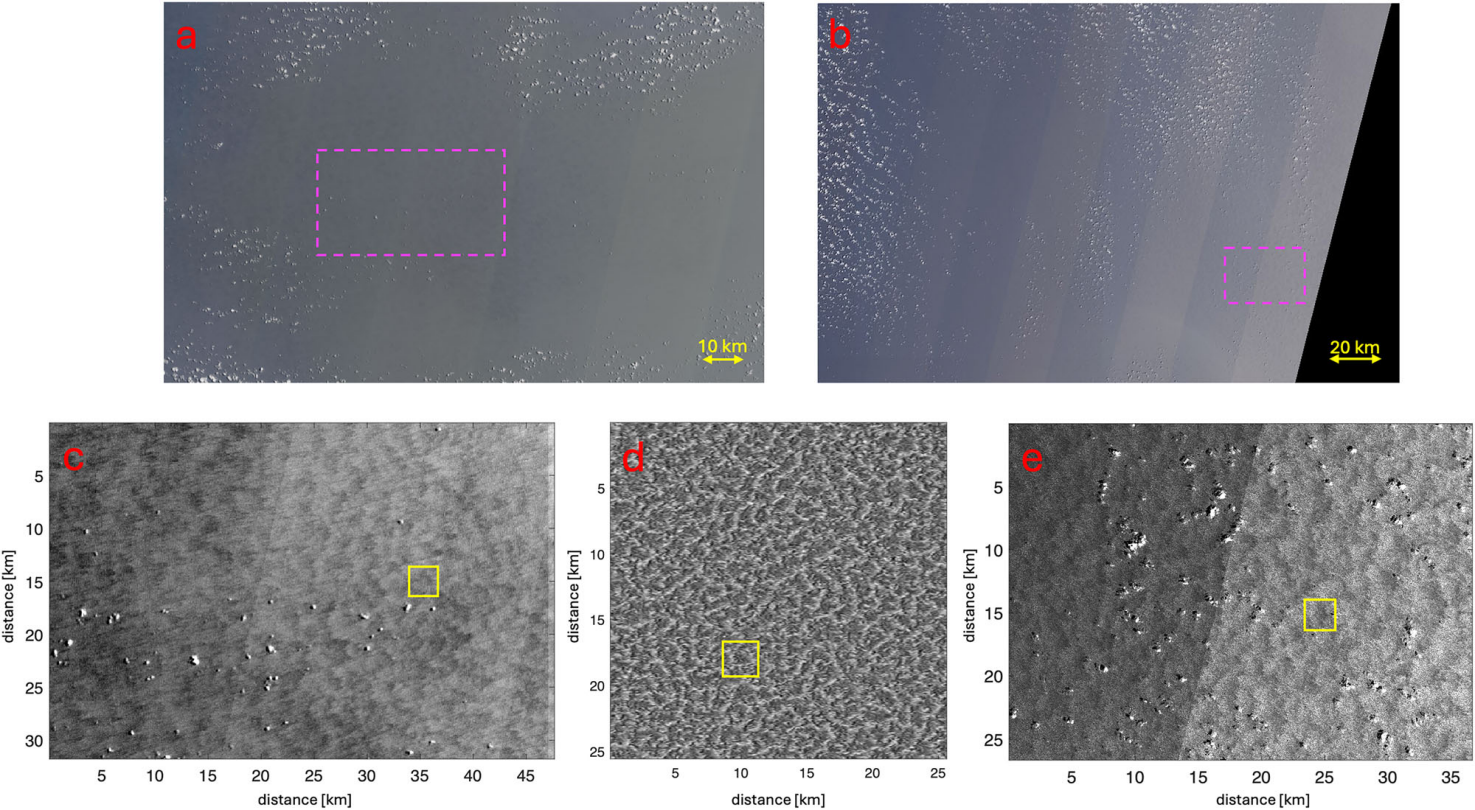
Two examples of the imprint of the subcloud dynamics as reflected in the sunglint. **a** MSI Sentinel-2 true color image of sparse TrCu clouds over the Caribbean Sea in the sunglint geometry (Lat 13.5, Lon -63.5), Aug 3, 2024, 10:30 mean local solar time and **b** shallow clouds over the Mediterranean Sea (Lat 32.1, Lon 28.9), Aug 6, 2025, 10:30 mean local solar time. Note that sunglint is stronger on the right (east) part of the images. **c** Zooming in and enhancing the blue channel (492 nm) to expand the low dynamic range of the sunglint over the area marked by the magenta rectangle in (**a**). A clear cellular structure, with a regular, organized pattern, appears on the surface covering the entire scene. **d** LES result: a snapshot of the simulated subcloud vertical velocity field at 240 m altitude. Note the similarity in scale and morphology between the LES-derived vertical-velocity pattern and the sunglint imprint over the sea surface. **e** Similarly to **c**, zooming in over the area marked by the magenta rectangle in (**b**), and enhancing the reflection of the blue channel. This example also shows that, similarly to the updraft field morphology (**d**), the entire water surface topography is composed of dense, organized, cellular structures. A yellow rectangle with a 2.5 km side is shown around a typical convective cell for **c**–**e**. Note that the clouds in **c**, **e** tend to form over the cells' edges (updrafts) and that in some cases there are enough clouds to mark the cell’s border. Note also that the image stripes with varying reflectance are part of the original data and are enhanced due to the stretched dynamical range in (**c**, **e**).

Since the typical diameter of the subcloud convective cells is ~1.5 km (see Supplementary Information Fig. S1), we explore high-resolution satellite images of the ocean’s surface of sparse TrCu fields in the sunglint directions, using 10m resolution data measured by the Multi-Spectral Instrument (MSI) on board the Copernicus Sentinel-2^47^. Figure 5 and Supplementary Information Figs. S3 to S6 show six examples of a sparse MBL convective cloud field. Two over the Caribbean Sea (Fig. 5a and Supplementary Information Fig. S3), two over the Mediterranean Sea (Figs. 5b and S5), one over the Atlantic Ocean near the Azores Islands, and one over the Pacific Ocean south of Mexico (both in Fig. S6). Panels 5c and 5e zoom in on the centers of the scenes (marked by the magenta boxes) and enhance the contrast of the lower reflectance range using only the blue channel (492 nm). The enhanced sunglint contrast is partitioned into dark circular spots, distributed almost uniformly, surrounded by brighter areas. For comparison, panel 5d shows an LES simulation snapshot of the updraft field (similar to Figs. 2 and 4) in the subcloud layer (at a height of 240 m). Note the similar pattern. Both the sunglint imprint and the simulated field display a dense, uniform cellular partition of the field, with cell sizes ranging ~1–2 km. To scale the cell sizes, a yellow rectangle measuring 2.5 km on a side marks a typical cell in each panel.

**Fig. 6:**
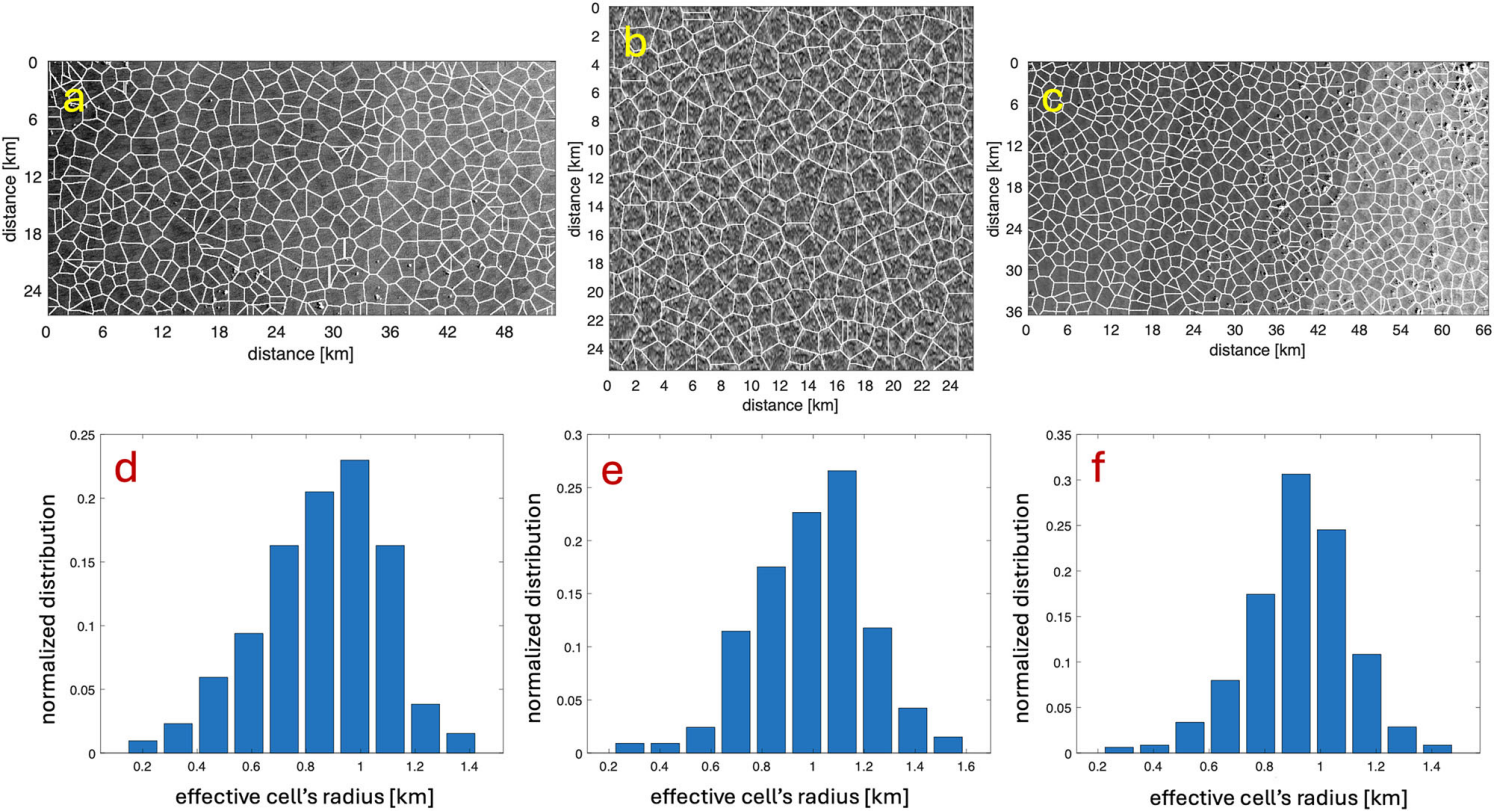
Quantification of the similarities between the subcloud’s vertical velocities, organized cellular patterns as shown from the LES to the imprint of the subcloud dynamics as reflected in the sunglint. The cellular partitions of the fields were detected using a watershed approach that traces and follows the connected bright areas (the subcloud layer updraft cell walls in the simulation). **a**–**c** Show the segmentation results of the Caribbean (Fig. 5a, c), the LES model (Fig. 5d), and the Mediterranean (Fig. 5b, e) cases, respectively. We note that a larger area was analyzed to get more statistics. **d**–**f** present the corresponding cell-size distributions for the Caribbean, the LES model, and the Mediterranean cases, respectively. The effective cell radius is calculated by approximating each cell’s area as a circle.

Note that the imprint exhibits a nearly domain-filling cellular texture, persisting even where no clouds are apparent. Moreover, the topology of dark and bright regions differs consistently, both in the imprint images and in the subcloud-layer LES fields. Bright regions, corresponding to updraft branches in the simulation, form a predominantly connected network, whereas dark regions appear mainly as isolated patches. This topological contrast enables objective cell identification and labeling, supports estimation of cell statistics, and facilitates quantitative comparison between the simulated organized convection and the sunglint-derived imprints. To segment and label individual cells, we apply a watershed-based segmentation approach^48^ to the filtered sunglint imprint and the model’s updraft field, delineating cell boundaries from the connected bright network and assigning isolated dark basins to individual cell interiors. Fig. 6 shows the segmented cellular partitions for all cases in Fig. 5, together with the corresponding cell-radius distributions (*r*_*cell*_, for a given cell area *A*, *r*_*cell*_ = *sqrt*(*A*/*π*)). The three distributions are highly similar: the mean cell radii are 0.86, 0.93, and 0.99 km, with standard deviations of 0.23, 0.21, and 0.22 km for the Caribbean (Fig. 5a, c), the Mediterranean (Fig. 5b, e), and the LES case (Fig. 5d), respectively. Such cell radii correspond to effective diameters of 1.85 ± 0.45 km. These values are broadly consistent with the ~1.5 km characteristic cell diameter inferred independently from the spectral analysis (Fig. S1), given the different definitions and the irregular, non-circular geometry of individual cells. As further illustrated in Supplementary Figs. S3 and S4, the cellular imprints differ markedly in both morphology and scale from ocean surface gravity waves, whose crest-to-crest wavelengths are typically ${\mathcal{O}}(10-100\,{\rm{m}})$ and whose signatures are elongated and oriented approximately orthogonal to the wind direction^49^.

## Discussion

Shallow marine convective clouds are often conceptualized as a localized process in which sparse thermodynamic perturbations lift parcels to the lifting condensation level (LCL), after which latent heating can sustain buoyant ascent. Recent studies have reported coherent structures below cloud base and suggested coupling between these structures and clouds aloft^24^. Here, we show that not only does the subcloud layer contain coherent structures, but that these structures are part of a dense, well-organized, steady mesh of convective cells.

In our previous work (Koren et al.^29^), we established a first-order departure from the “random perturbation” picture by showing that sparse trade cumulus clouds form within a well-organized, stable, and densely packed cellular convective field that operates continuously, largely independent of visible cloud presence, and that this steady cellular pattern predicts where clouds recur. This provided the first quantitative link between near-deterministic, highly organized boundary-layer dynamics and apparently sparse, intermittent clouds.

Here, we go substantially further by resolving and mechanistically linking two distinct dynamical regimes: steady, organized subcloud convection and intermittent, sparse, short-lived clouds. We do so by resolving the system as a two-layer structure with different dynamical behavior: (i) a subcloud layer that hosts a regular, dense, steady, open-cell-topology convective machinery whose characteristic cell lifetime is comparable to the cloud-field lifetime, and (ii) a cloud layer whose vertical-velocity field is intermittent, with broader tails and localized plumes of strong updrafts associated with cloud formation.

Using passive Lagrangian tracers, we show that this lower-layer machinery is not merely present but continuously operating and largely closed. Most trajectories recirculate within subcloud cells, while only a small fraction penetrates into the cloud layer (about 15%). Crucially, we identify the coupling pathway. Rare overshooting/penetrating events preferentially originate along the subcloud updraft walls, and the resulting momentum, heat, and moisture leakage into the conditionally unstable layer aloft acts as the perturbation that triggers plume and cloud initiation, consistent with the observed alignment between cloud-layer strong-updraft regions and the subcloud cell edges.

Using a Lagrangian framework that removes mean subcloud advection, we transport each intermittent cloud-layer plume to a common reference time (e.g., *t*_0_) and accumulate the corrected fields (Fig. 4). In this Lagrangian frame, the cloud-layer updraft “hot spots” no longer appear random: they reconstruct an almost stationary cellular pattern that mirrors the steady subcloud lattice, revealing that cloud recurrence is anchored by the persistent subcloud organization despite strong intermittency aloft.

Such subcloud dynamics and their coupling to the cloud formation mechanism shed new light on TrCu fields. These results reframe non-precipitating trade cumulus formation as a system in which a dense, deterministic, long-lived subcloud convective backbone intermittently seeds a short-lived, sparse cloud-layer response, rather than clouds being primarily isolated outcomes of local, short-lived near-surface perturbations.

These findings provide a physically grounded basis for linking subcloud dynamics to cloud occurrence and motivate organization-aware parameterizations that connect steady boundary-layer dynamics to cloudiness and radiative effects across marine boundary-layer regimes.

## Methods

### LES model simulations

We used SAM v6.10 - a three-dimensional, non-hydrostatic, anelastic large-eddy model that explicitly resolves boundary-layer turbulence and cloud dynamics, to reproduce the classic BOMEX trade-wind cumulus case (June 1969, 13^∘^ N, 59^∘^ W)^18,50^. The BOMEX case study remains the standard prototype for “sugar” TrCu conditions and has been shown to be representative of trade-wind cloud statistics across the Atlantic and Pacific basins^30^.

The computational domain was 25.6 × 25.6 × 4.0 km^3^ with periodic lateral boundaries and a Rayleigh-damped lid above 3.5 km. Grid spacings were 50 m horizontally and 40 m vertically (100 levels). Large-scale subsidence, advective temperature-moisture tendencies, and a fixed SST of 300 K followed the intercomparison specification of Siebesma et al.^18^. The model was integrated for 8 h with a dynamical time step of 1 s; the first 2 h were discarded as spin-up.

Microphysical processes were handled by the Spectral Bin Microphysics (SBM) scheme^32^, which explicitly solves warm-phase processes, including nucleation, condensational growth, collision-coalescence, breakup, and sedimentation, on 33 CCN and droplet bins. We prescribed a uniform cloud-condensation-nuclei concentration of 500 cm^−3^, yielding non-precipitating “sugar” cumuli throughout the run.

### Lagrangian frame correction and advection estimate

The BOMEX case study prescribes a height-dependent horizontal wind (advection) field. In principle, one could apply a distinct Lagrangian correction at every level by simply subtracting the averaged advection at each timestep. In practice, however, turbulent mixing, frictional drag, and internal mesoscale dynamics cause cloud elements to depart from the mean flow, making an a priori height-by-height correction unreliable.

Instead, we estimate the Lagrangian displacement from the data themselves, following the cross-correlation ("pattern-matching”) approach of ref. ^36^. Let *τ*_morph_ denote the characteristic time over which cloud morphology changes appreciably in a chosen subdomain, and let *Δt* be the snapshot interval. Provided that *τ*_morph_ ≫ *Δt*, we may regard successive images as almost rigid translations of one another. The optimal horizontal shift ***δ*** is obtained by maximizing the two-dimensional cross-correlation (or, equivalently, minimizing the mean-square difference) between a function *η*(*I*_*t*_) of consecutive snapshots *I*_*t*_ and *I*_*t*+*Δt*_. *η*(*I*_*t*_) could be the snapshot itself or any gradient function of it. In practice, the normalized error surface exhibits a sharp minimum, so ***δ*** is well defined. We compute ***δ*** for every time step and subtract it to obtain Lagrangian-corrected fields.

This procedure has been successfully applied to both satellite imagery and large-eddy simulations^28,36,48^. As we demonstrate in the paper, the relatively short-lived plumes in the cloud layer are likely to be initiated by perturbations from the subcloud layer. Therefore, their overall advection is coupled to the subcloud layer, as revealed by the match between the cloud and the subcloud layers (Fig. 4) following a subcloud Lagrangian correction for both.

## Supplementary information

Supplementary information

## Data Availability

The Lagrangian corrected data of the simulated cloud LWP and vertical velocities are available in an online repository at 10.34933/c2ff596a-eb3b-4f77-882d-b633ef971ce5.
