# Supplementary material for: Sunglint imprints of steady subcloud cells anchoring intermittent trade cumulus
Source: NPJ Clim Atmos Sci. 2026 May 12;9(1):159. doi: 10.1038/s41612-026-01427-3 (PMC13391368; doi:10.1038/s41612-026-01427-3)
Supplement: Supplementary file 1 — Supplementary information [file 41612_2026_1427_MOESM1_ESM.pdf]

Supplementary Information for
Sunglint imprints of steady subcloud cells anchoring intermittent trade cumulus

Ilan Koren[1*], Orit Altaratz[1], Yeal Arieli[1], Bar Moisa[1]

[1]Department of Earth and Planetary Science, Weizmann Institute of Science, Rehovot, Israel.

[*]Corresponding author. Email: ilan.koren@weizmann.ac.il

This PDF file includes:

Figures S1 to S6

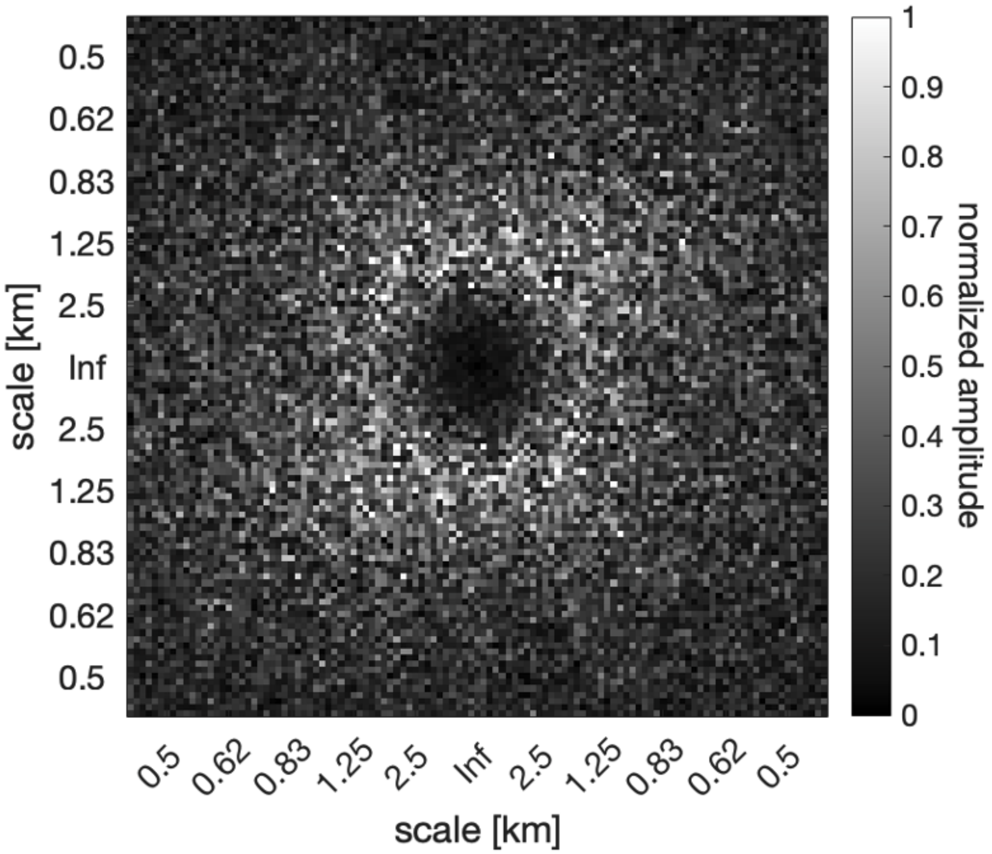

Figure S1: **Spectral Analysis of the updraft fields.** Two-dimensional absolute Fourier spectrum (FFT) of the subcloud updraft field w_{240} (Fig. 2a). Note the periodicity units. The apparent rings in the spectral analysis indicate the characteristic cell sizes of $\sim$ 1.5 km.

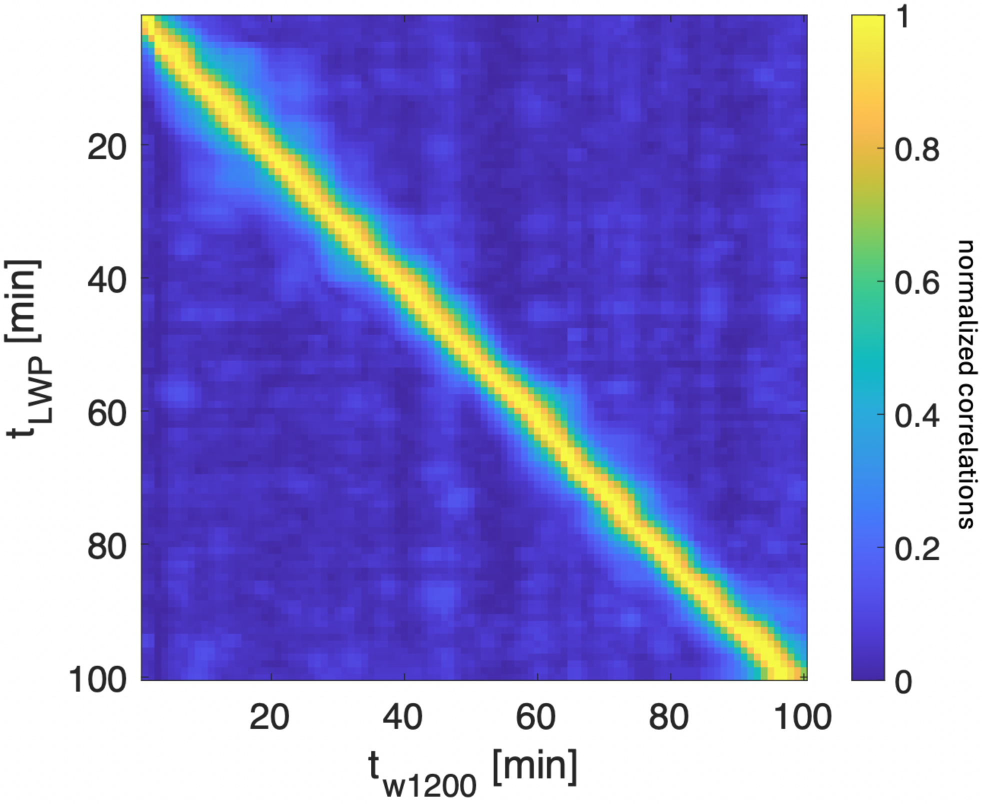

Figure S2: **On the link between clouds and vertical velocities in the cloud layer.** In this paper, we focus on the velocities as a direct measure of the dynamics. There is a direct link between the vertical velocity field and cloud formation. Convective clouds form within updrafts. To demonstrate this link, we correlate the integrated liquid water path field (LWP), which measures the total column amount of condensate within the clouds, to the vertical velocities in the middle of the cloud layer w_{1200}. For each time t_{LWP}, we compute the correlation between the LWP snapshot and w_{1200} at all times t_w, yielding the correlation matrix $C(t_{LWP}, t_w)$ shown for a 100-min segment in the middle of the simulation. The pronounced ridge near the diagonal ($t_{LWP} \approx t_w$) indicates that LWP patterns align most strongly with the contemporaneous updraft structure, consistent with cloud condensate occurring within updrafts. The rapid weakening of correlation away from the diagonal indicates that this alignment decorrelates quickly with increasing time lag $|t_w - t_{LWP}|$, reflecting the short-lived, intermittent nature of the cloud-layer field.

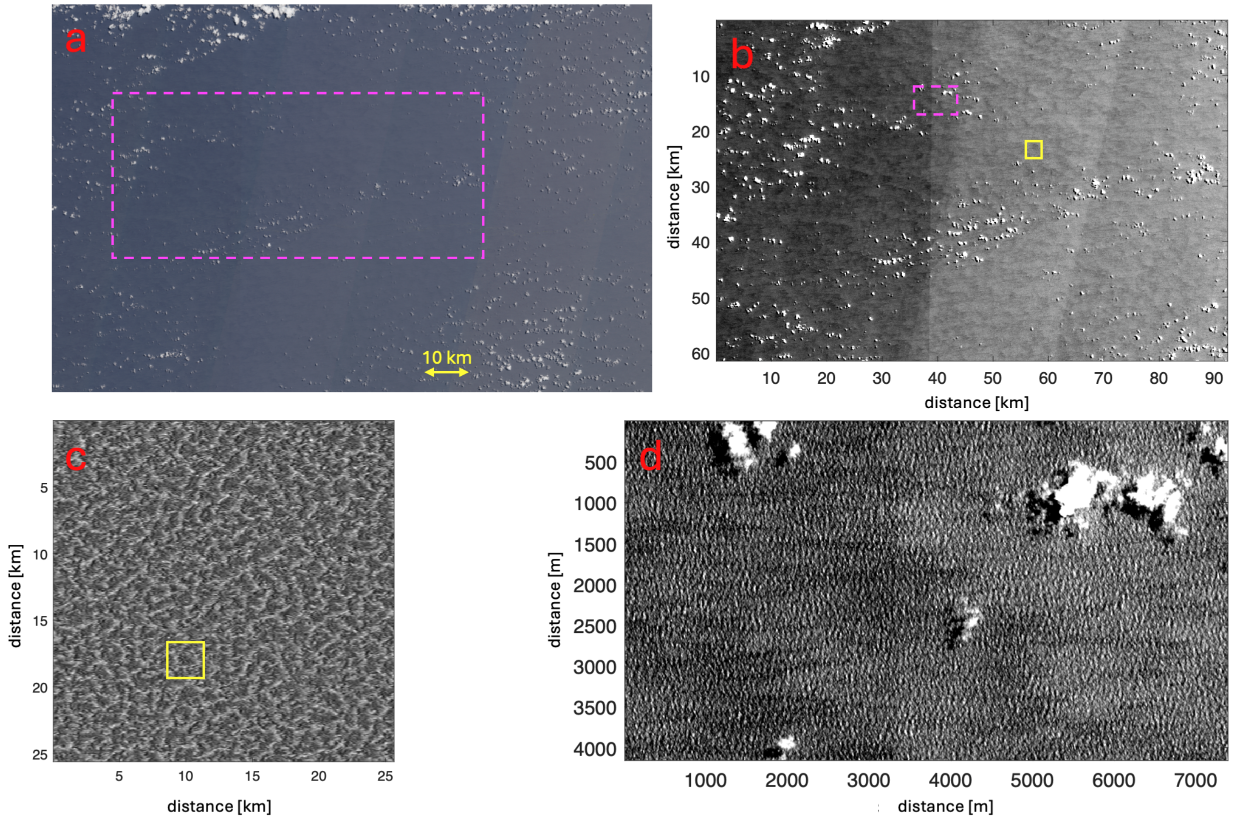

Figure S3: **Subcloud dynamics imprint's scale vs. the scale of waves in the ocean.** a) An additional case of MSI Sentinel-2 true color images of sparse TrCu clouds is shown. The field forms over the Caribbean Sea and is shown in the sunglint geometry. The image is centered at Lat 18, Lon -55, on June 20, 2024, at 10:30 mean local solar time. b) Zooming in and enhancing the blue channel (492 nm) to expand the low dynamic range of the sunglint over the area marked by the magenta rectangle in panel a. Note how the cellular structure appears on the surface, covering the entire scene. c) Similarly to the case shown in Fig. 5 in the main text, a snapshot of the model's vertical velocity field in the subcloud layer (w_{240}) is shown. To compare the cells' morphology and scale, a yellow rectangle with a 2.5 km side is shown around a typical convective cell, both in the enhanced image in panel b and on the snapshot of the model's vertical velocity field in panel c. d) Zooming in over the area marked by the magenta box in panel b, showing the surface wave patterns. It shows that the wave crests are aligned in the north-south direction, orthogonally to the surface easterlies (trade) winds. Note the differences between the scale of the waves and the scale of the cellular imprint, in which their downdraft centers are shown as darker gray areas in the zoom-in example.

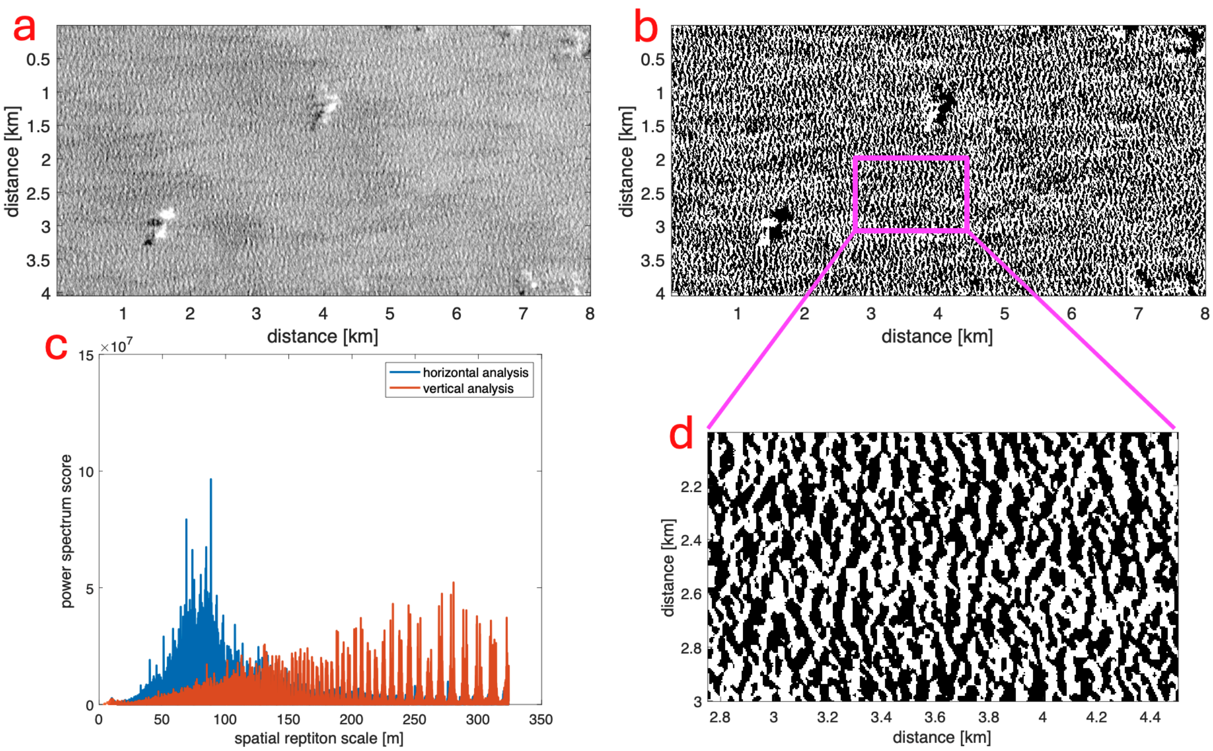

Figure S4: **Spectral analysis of the gravity waves.** To further analyze the scale of the gravity waves, we focus on the high-resolution sunglint image shown in Fig. S3. To minimize cloud contamination, panel (a) shows the lower portion of Fig. S3b, where cloud cover is minimal. The dynamic range is further stretched to encompass the gravity wave range. The MSI Sentinel-2 image in the glint suffers from brightness gradients driven by changes in the sun-satellite angle. Moreover, the dynamical imprint of the lower atmosphere is evident as subtle changes in the surface brightness, making the analysis of smaller-scale gravity-wave patterns challenging. To unify the gravity wave reflectance range, we calculated the large-scale average brittleness map using a low-pass filter (500 m scale) and extracted this average from the original brightness map. Panel (b) shows the gravity wave mask calculated after reducing the background gradients. Panel (d) shows a zoom in on the marked magenta rectangle, clearly showing the elongated waveform. To quantify the repetition scale, we calculated the power spectrum along the trade-wind (x-axis) and cross-wind (y-axis) directions of the image. Panel (c) presents the two power spectra. The spectrum in the along-wind direction (blue), which crosses the wave crests, shows a clear peak centered around 80 m, whereas the spectrum in the cross-wind direction (red) shows no distinct preferred scale.

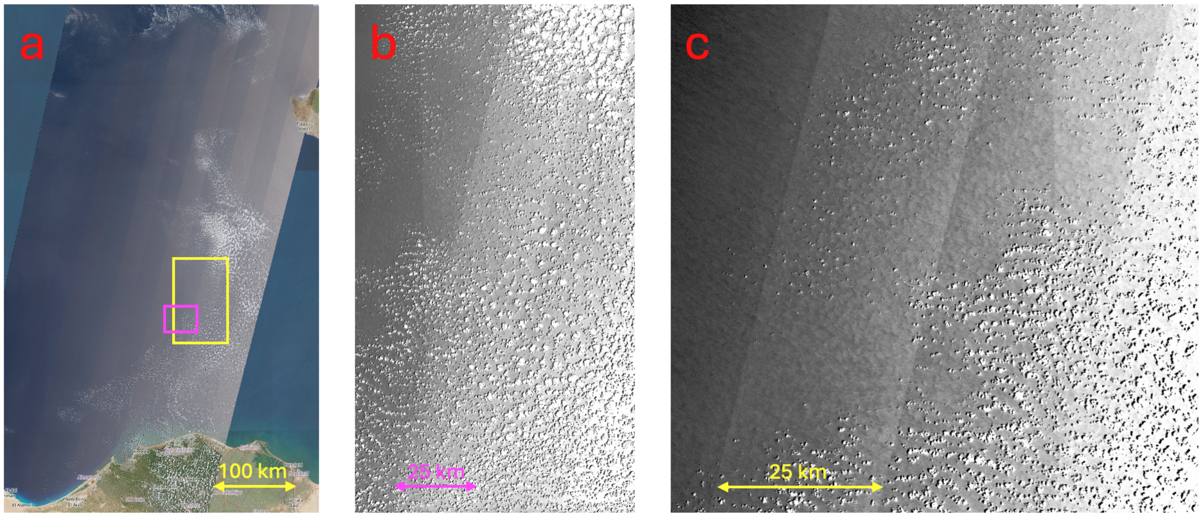

Figure S5: **Subcloud dynamics over the Mediterranean in three scales** a) A boundary layer cloud field southwest of Cyprus. A true color MSI Sentinel-2 image centered at Lat 32, Lon 25, on July 14, 2025, at 10:30 mean local solar time. b) Zooming in and enhancing the contrast around the sunglint's dynamic range over the area marked by the yellow rectangle in panel a. c) Further zooming in over the area marked by the magenta rectangle in panel a. Note the cellular patterns shown over the ocean below the cloud field.

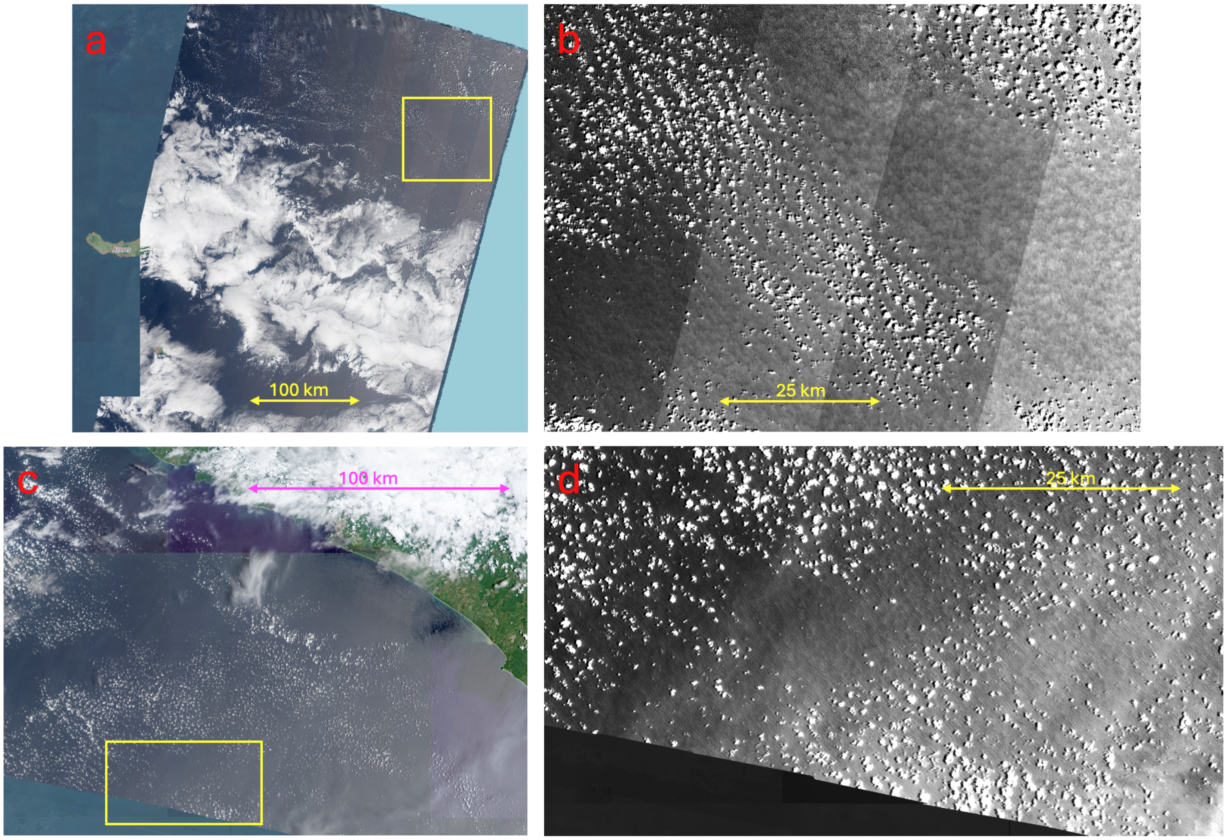

Figure S6: **Subcloud dynamics examples near the Azores Islands and south of Mexico** a) A true color MSI Sentinel-2 image of clouds east of the São Miguel Island, centered at Lat 32, Lon 25, on July 20, 2025. b) Zooming in and enhancing the contrast around the sunglint's dynamic range over the area marked by the yellow rectangle in panel a. c) A true color MSI Sentinel-2 image of scattered clouds southwest of Mexico City. d) Zooming in and enhancing the contrast around the sunglint's dynamic range over the area marked by the yellow rectangle in panel c. Note the cellular patterns shown over the ocean below the cloud fields.
